# Low-Complexity Multi-Size Circular-Shift Network for 5G New Radio LDPC Decoders

**DOI:** 10.3390/s22051792

**Published:** 2022-02-24

**Authors:** Tuy Tan Nguyen, Tram Thi Bao Nguyen, Hanho Lee

**Affiliations:** Department of Information and Communication Engineering, Inha University, Incheon 22212, Korea; tuynguyen@inha.ac.kr (T.T.N.); baotram137@gmail.com (T.T.B.N.)

**Keywords:** 5G New Radio, decoder architectures, LDPC code, switch network

## Abstract

This paper presents a low-complexity multi-size circular-shift network (MCSN) structure for 5th-generation (5G) New Radio (NR) quasi-cyclic low-density parity-check (QC-LDPC) decoders. In particular, a fine-coarse approach-based multi-size cyclic shift network, which decomposes the cyclic shift size into fine part and coarse part, is introduced. The proposed MCSN structure is composed of a pre-rotator performing the fine part of the cyclic shift, and a main rotator executing the coarse part of the cyclic shift. In addition, a forward routing circular-shift (FRCS) network, which is based on the barrel shifter and the forward routing process is presented. The proposed switch network is able to support all 51 different submatrix sizes as defined in the 5G NR standard through an efficient forward routing switch network and help reduce the hardware complexity using a cyclic shift size decomposition method. The proposed MCSN is analyzed, and indicates a substantial reduction in the hardware complexity. The experimental results on TSMC 65-nm CMOS technology show that the proposed MCSN structure for 5G NR LDPC decoder offers an area saving up to 56.75% compared to related works in the literature.

## 1. Introduction

Low-density parity-check (LDPC) codes [1], which were first proposed by R. Gallager in the early 1960s and rediscovered by MacKay and Neal [2] in 1996, have received significant attention thanks to their powerful error correction capability. Recently, LDPC codes have been adopted in the enhanced Mobile Broad Band (eMBB) as the error correction coding scheme for the fifth generation (5G) data channel [3]. The 3rd Generation Partnership Project (3GPP) introduced two rate-compatible base graphs, BG1 and BG2 [4], to support the rate compatible and scalable data transmission. Accordingly, several studies have been conducted on the 5G New Radio (NR) LDPC codes [5,6,7,8]. In [5], an adaptation of the MinSum decoders for the LDPC used in the eMBB scenario in the 5G mobile networks is proposed. In [6], a hardware-friendly LDPC decoding schedules for 5G hybrid automatic repeat request applications is presented. An algebra-assisted method for constructing 5G LDPC codes can be found in [7]. An efficient encoding method and a high-throughput low-complexity encoder architecture for the 5G NR standard is presented in [8].

In recent years, research on LDPC codes has centered around structured LDPC codes known as the quasi-cyclic low-density parity-check (QC-LDPC) codes [9,10,11,12,13], which have demonstrated great advantages over other types of LDPC codes in terms of hardware implementation and excellent error performance over noisy channels. In QC-LDPC codes, the parity-check matrix consists of either cyclic permutation submatrixes or zero matrixes of the same size, determining the interconnection between the check node processing units (CNUs) and variable node processing units (VNUs). The interconnection network for the QC-LDPC codes is highly structured and can be characterized by the submatrix size and the circular-shift value of each cyclic permutation submatrix. The decoding process of a QC-LDPC code requires circular-shift network (CSN) because of its cyclic characteristic. However, a QC-LDPC decoder for emerging wireless communication systems must support multiple code rates, various block lengths, and different submatrix sizes. The QC-LDPC codes adopted by the WiMAX standard (IEEE 802.16e) [14] supports 19 different submatrix sizes with four distinct code rates and six different class codes (distinct distributions of the number of variable nodes per column of check nodes per row). The submatrix sizes vary from 24 to 96. In the 5G NR LDPC code specification, 51 different lifting sizes are defined, and there are eight different permutation matrix designs per base graph [4]. For this reason, the switch network between CNUs and VNUs should be reconfigurable to support any submatrix sizes defined in the standards of applications. A switch network with varying sizes is called a multi-size circular-shift network (MCSN).

Over the past few years, various works have been introduced in order to improve the signal congestion and hardware complexity of the MCSN for QC-LDPC decoders [15,16,17,18,19]. Benes network [15], which is capable of routing any Z×Z permutation where *Z* is a power of 2, is a widely used structure. Benes network has been proven to be an optimal non-blocking permutation network. The main drawback of this method is its long critical path and high complexity, which makes it less efficient for implementation with the large size of submatrixes. One of the simplest approaches is assigning a multiple-input multiplexer (MUX) to each output. A MUX is assigned to an output, and each MUX has as many inputs as the number of input data items of the MCSN. This method suffers from high complexity since very wide-input MUXs is required. To overcome the overhead incurred by wide-input MUXs, Rovini [16] proposed a pre-rotator structure, in which small pre-rotators reduce the input width of the MUXs considerably. Furthermore, many other structures have been presented such as: rotator-in-series (RIS) structure [17], rotator-in-parallel (RIP) structure [16,18], and fine-coarse (FC) structure [19]. However, the switch network structures in [15,16,17,18,19] are not suitable for 5G NR LDPC decoders where the submatrix sizes cover a very wide range from 2 to 384. To overcome the limitations of those approaches, this paper considers the design of low-complexity multi-size circular-shift network for 5G NR LDPC decoders, which requires significantly low hardware complexity compared to the conventional architectures. To the best of our knowledge, this is the first work proposing a low-complexity MCSN for the 5G NR LDPC decoders in the literature.

This paper targets the design of a novel low-complexity MCSN structure for 5G NR LDPC decoders using the fine-coarse approach. The proposed MCSN architecture can support all the cyclic shifts for all submatrix sizes defined in the 5G NR standard. The simulation results show that the area of the proposed structure is reduced more than 18% compared to the existing works. Therefore, our study provides a promising multi-size cyclic-shifter design for 5G NR LDPC decoders.

The rest of this paper is organized as follows. Section 2 gives a brief overview of the characteristics of 5G NR LDPC codes and outlines the conventional LDPC multi-size cyclic-shifter structures in the literature. A novel low-complexity MCSN architecture is described in Section 3. Section 4 presents the implementation results and comparison. Finally, conclusions are drawn in Section 5.

## 2. Multi-Size Circular-Shift Network for 5G NR LDPC Decoder

### 2.1. 5G NR LDPC Characteristics

QC-LDPC codes play a crucial role in 5G communications. In the 3GPP standard meeting, QC-LDPC codes were accepted as the channel coding scheme for 5G eMBB data channel. The brief structure of NR LDPC base graph is illustrated in [8]. In particular, the columns of the base graph are partitioned into three parts: information columns, core parity columns, and extension parity columns. The rows of the base graph are divided into two parts: core check rows, and extension check rows. Specifically, the base matrix consists of five submatrixes: (1) submatrix A is reserved for systematic bits; (2) submatrix B, a square matrix with dual-diagonal structure, corresponds to the first set of parity bits; (3) submatrix O is an all-zero matrix; (4) submatrix C corresponds to single parity-check (SPC) rows; and (5) an identity matrix I corresponds to the second set of parity bits. The combination of submatrix A and submatrix B is defined as the kernel, and three submatrixes O, I and C are called extensions. This code structure is similar to the Raptor-like extension as described in [20].

The 3GPP has agreed to consider two rate-compatible base graphs, BG1 and BG2, which have a similar structure for the channel coding. While BG1 is designed for larger block lengths (500≤K≤8448) and higher rates (1/3≤R≤8/9), BG2 is targeted for smaller block lengths (40≤K≤2560) and lower rates (1/5≤R≤2/3). The exact usage of base graphs and the definition of the two matrixes can be found in the 5G NR standard specification [4].

### 2.2. Multi-Size Circular-Shift Network

In addition to computation units, the switch network is an essential element which occupies significant portion of the LDPC decoder area. In QC-LDPC codes, the parity-check matrix is composed of cyclic permutation submatrixes that determine the interconnection network between the CNUs and VNUs. Accordingly, the decoder needs to circularly shift an array of *Z* messages where *Z* being the size of the submatrix, also referred to as block size. QC-LDPC codes simplify the switch network by a great deal as the requirement of all random shifting (or permutation) is eliminated and only cyclic shifting is required.

In many modern communication standards, the LDPC codes are defined for different codeword lengths and block sizes $Z_{k}$, with 0≤k≤K, where *K* is the number of admissible sizes. Therefore, an MSCS network, which is capable of circular shift over an arbitrary number of data, is mandatory. An MSCS network block rotates a specified amount of data. If *Pw*-bit data $\left(d_{0},d_{1},d_{2},\cdots ,d_{P-1}\right)$ are inputted with a shift size *Z* and circular-shift amount *c*, the first *Z* input data are rotated by *c* to produce the output $\left(d_{c},d_{c+1},\cdots ,d_{Z-1},d_{0},d_{1},\cdots ,d_{c-1}\right)$. The output data beyond the specified size of *Z* are redundant. The sizes and shift amounts are determined by the adopted QC-LDPC codes.

**Figure 1:**
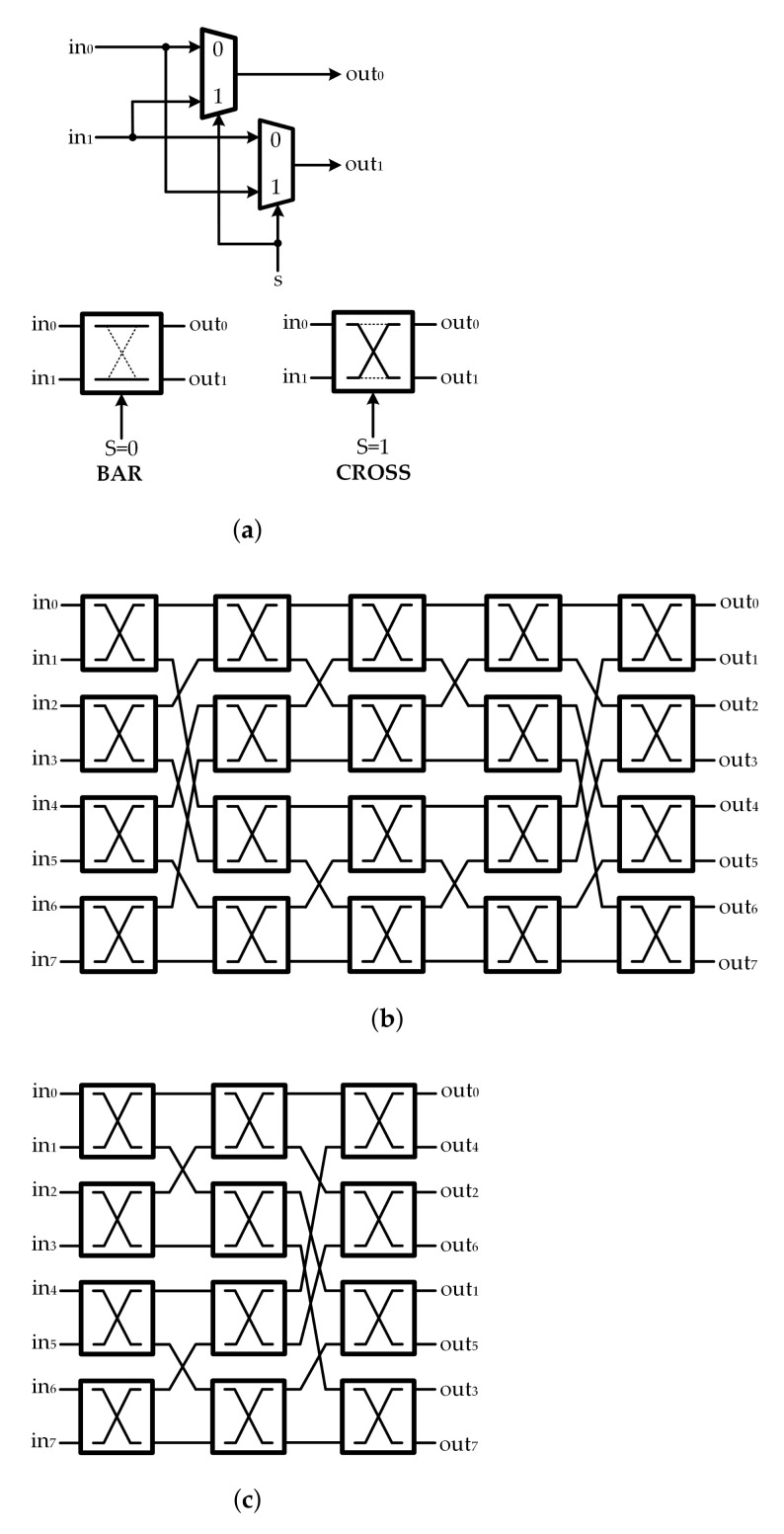
(**a**) Benes network 2×2 switch; (**b**) Benes network when *P* = 8; (**c**) Banyan network when P=8.

Generally, the Benes network is a very well-known non-blocking multistage interconnection network. A P×P Benes network is capable of performing any desired permutation over *P* data units, where the input size *P* of the Benes network is a power of 2. The network comprises of $\left(2log_{2}P-1\right)$ stages of 2×2 switches. Each switch element consists of two 2-input MUXs and can operate either in the BAR or in the CROSS state, as depicted in Figure 1a. An example of a Benes network with *P* = 8 is shown in Figure 1b. The switch network designed for QC-LDPC decoders is generally optimized for a given size of permutation matrix with the Benes network. To properly control all the switch units and realize the desired permutation result, the control signals for all 2×2 switches must be provided. Those control signals are pre-computed and stored in a dedicated LUT. However, as the submatrix size increases, the required size of LUT becomes a dominant area consuming factor. Therefore, the Benes network with a large size of submatrix is inefficient as it suffers from significant hardware overhead. Furthermore, Benes network offers a limited application, as the number of inputs should be a power of 2.

For the QC-LDPC decoders, switch networks are only required to implement the circular shifts. It was shown that only the first or last $log_{2}P$ stages of the *P* input Benes network are needed to perform all cyclic shifts for *P* inputs [21]. Therefore, the implementation of the half or reduced Benes network referred to as Banyan network [22] in Figure 1c, is a preferable switching network structure. When the submatrix size is not a power of 2, it is possible to use an $P^{\prime }\times P^{\prime }$ Banyan network where $P^{\prime }=2^{\left\lceil log_{2}P\right\rceil }$. However, an additional multiplexing stage must be carried out either at the switch network input or output. In recent years, many techniques were proposed to modify the Benes network to make it more efficient for the reconfigurable QC-LDPC decoders [15].

**Figure 2:**
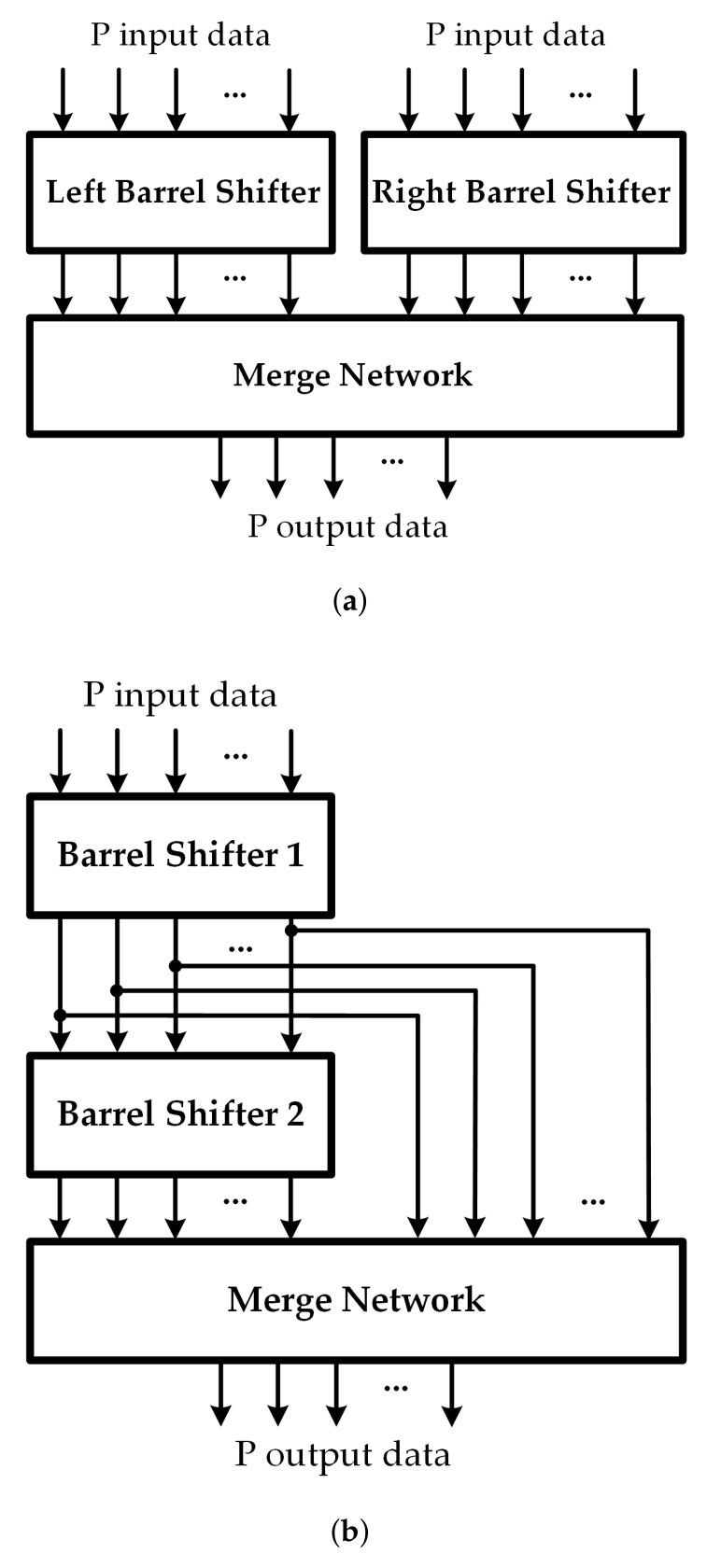
(**a**) RIP structure; (**b**) RIS structure.

The MSCS network structures can be implemented with typical barrel rotators. The structures exploit two barrel rotators which can be placed in parallel [23] or in series [17], as shown in Figure 2a,b, respectively. The output data of the MSCS network are combined from the outputs of two barrel rotators. In the Rotator-In-Parallel (RIP) structure [23], the left rotator performs the left shift by *c* to the input data, and the right rotator performs the right shift by P−c to the input data. After that, the obtained results from the barrel rotators are combined by MUXs to become the output data. In the Rotator-In-Series (RIS) structure, the first barrel rotator, rotates the input data by *c*. The result obtained from the first barrel rotator is then rotated by the second barrel rotator in the same direction by P−Z[17]. In addition, the output data are selected from the outputs of the barrel rotators and then combined by MUXs to become the output data. One of the most straightforward ways to employ an MSCS network block would be using a MUX network. An *P*-to-1 (*w*-bit) MUX can be used to connect *P* inputs to an output since the output data can be selected from *P* input data. The main drawback of this structure is that many wide-input MUXs are required. These wide-input MUXs are normally implemented by cascading a lot of 2-to-1 MUXs.

**Figure 3:**
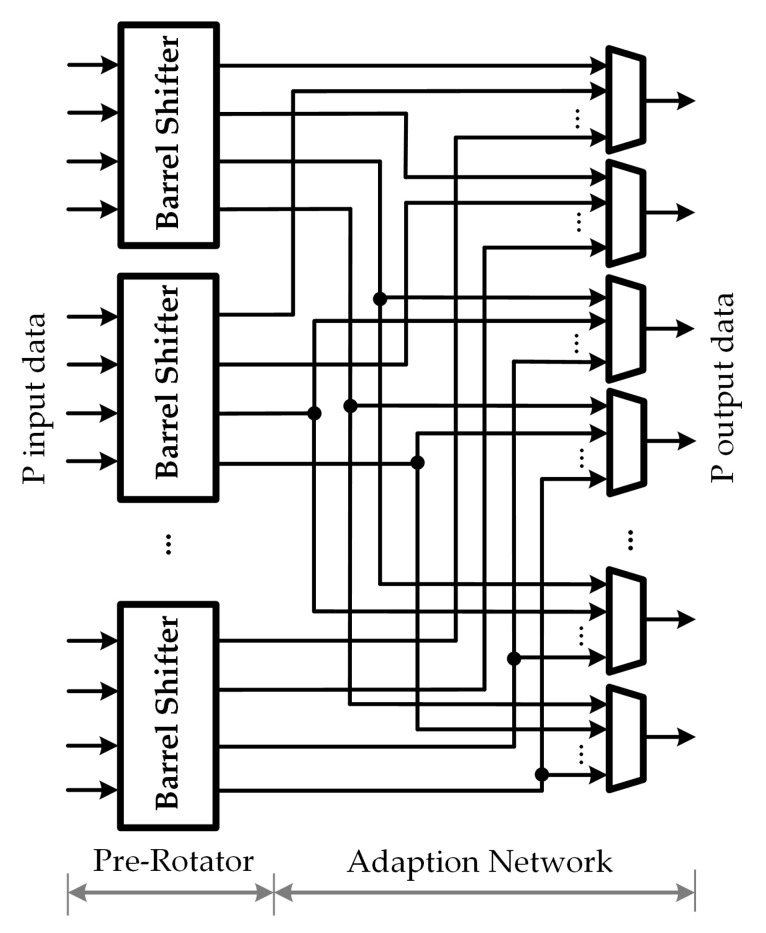
Pre-rotator and MUX network structure.

To improve the performance of the MUX network structure, Rovini [16] introduced a pre-rotator structure exploiting the size-common-divisor property, as shown in Figure 3. The input data are rotated by pre-rotators so that the amount of input data is the greatest common divisor (GCD) of all block sizes before being processed by a MUX network. More precisely, with the maximum block size *P*, the MSCS network is composed of $P_{g}$ *g*-input pre-rotators and additional stage of *P* $P_{g}$-to-1 MUXs, also referred to as adaptation network (AN), where *g* is the GCD and $P=g\times P_{g}$. This structure substantially reduces the MUX input width from *P* to *g*. However, the MUX network still occupies a significant area. Specifically, the equivalent number of 2-to-1 MUXs is $\left[\left\lceil log_{2}g\right\rceil \times P+\left(P_{g}-1\right)\times P\right]$, where the first term and the second term are for the pre-rotators and the MUX network, respectively. In [19,24], the MUX network in the AN is substituted with the Benes network and the RIP structure, respectively, to enhance the complexity of the MSCS network block.

The previous MSCS network structures are proposed to support multiple submatrix sizes in the QC-LDPC decoders. However, previously proposed structures are not suitable for 5G NR LDPC decoders where the submatrix size varies largely from 2 to 384. The considerable variation in submatrix sizes would result in the high inefficiency in hardware use and the high complexity in the interconnecting network in conventional switching network structures. To overcome the limitations of existing MSCS network structures, the design considered in this paper, referred to as low-complexity multi-size circular-shift network for 5G NR LDPC decoders, requires significantly low hardware complexity compared to conventional structures.

## 3. Proposed Low-Complexity Multi-Size Circular-Shift Network for 5G New Radio LDPC Decoder

### 3.1. Modified Lifting Size Table for 5G New Radio LDPC Codes

**Figure 4:**
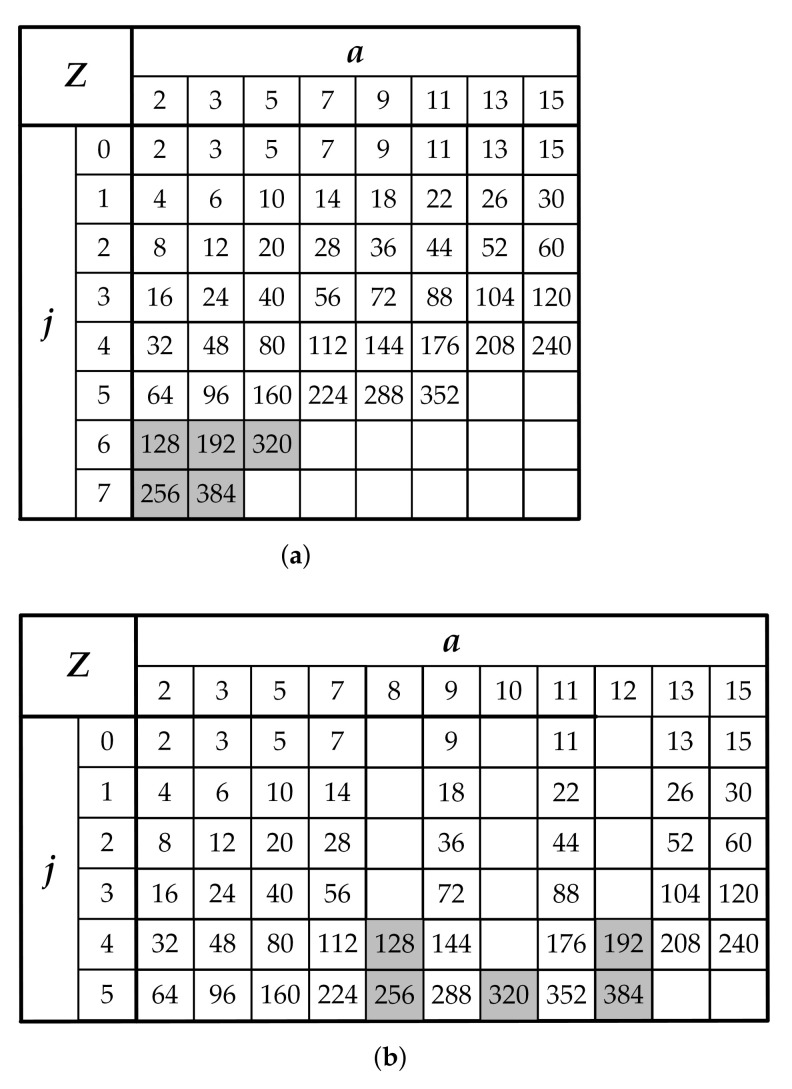
(**a**) Lifting size *Z* table; (**b**) Modified lifting size *Z* table for standard 5G LDPC codes.

LDPC codes for the 5G NR wireless communication standard are defined for 51 different lifting sizes *Z* as shown in Figure 4a. The set of lifting sizes *Z* supported for the base graph are all values of the form $Z=a\times 2^{j}$ for a=2,3,5,7,9,11,13,15 where 0≤j≤7 and includes all such *Z* distributed over the range from 2 to 384. The lifting sizes *Z* can be organized into eight sets, one for each value of *a*. To make it more convenient for our proposed MSCS network structure, the lifting size table is efficiently modified as shown in Figure 4b. Precisely, the two rows for j=6 and 7 are removed from the modified table. Instead, three new columns for a=8, 10, and 12 are created in the table. Accordingly, the value of lifting sizes Z=128, 192, 256, 320, and 384 are sufficiently reallocated into new positions in the modified table.

### 3.2. Structure of Forward Routing Network

**Figure 5:**
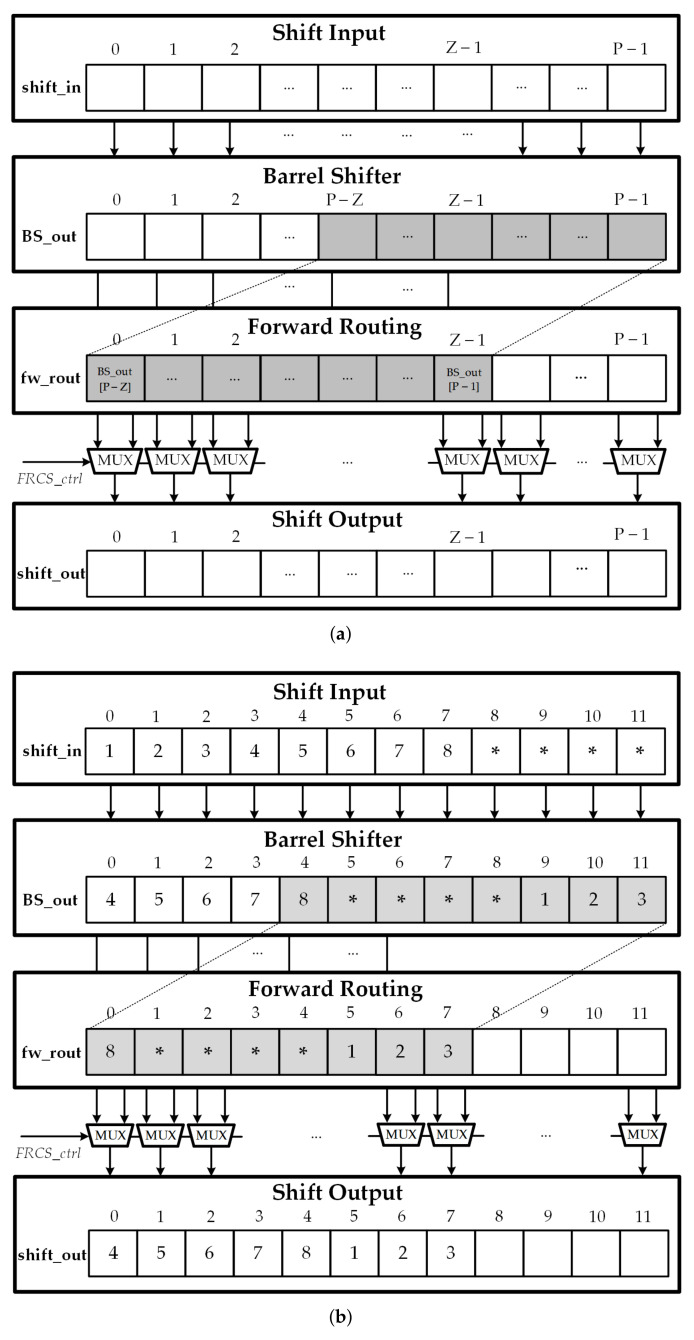
(**a**) FRCS network structure; (**b**) An example of FRCS network with Z=8, P=12 and c=3.

In this section, an efficient reconfigurable message passing network based on the barrel shifter and an additional forward routing stage is presented. Please note that the maximum input size that can be supported by the circular-shift network is *P*, the block size of the input data for the circular shift is *Z*, and the value of the required circular shift is *c*, where 0≤c<Z≤P. The structure of the Forward Routing Circular-Shift (FRCS) network is illustrated in Figure 5a, which includes 3 stages. The first stage is the barrel shifter, the second stage is the forward routing module, and the final stage is the selection scheme. The barrel shifter with input size *P* in the first stage can be easily constructed from $\left\lceil log_{2}P\right\rceil$ stages of 2-to-1 MUXs. The barrel shifter circularly shifts the input message shift_in according to the specific shift amount *c*. The control signals for the barrel shift network can be easily determined from the value of the shift amount *c*. In the next step, the forward routing module simply takes the value of the (P−Z)th to (P−1)th shifted message BS_out as input data and fetches into next stage. The shifted message from the barrel shifter BS_out and the forward routing message fw_out is fed into the final stage to generate the expected output. The selection stage is in charge of selecting a signal from the barrel shifter or the forward routing network and routing a proper signal to the output. In the selection stage, the expected output message shift_out is determined by a MUX network where the control signal $FRCS_{ctrl}$ is defined as follows:

(1) $$FRCS_{ctrl}\left[i\right]=\left\{\begin{array}{cc} 0 & \;\mathrm{if}\;0\leq i<Z-c,\\ 1 & \;\mathrm{if}\;Z-c\leq i<Z. \end{array}\right.$$

According to the controller design, the final output messages shift_out[i] comes from the forward routing module if 0≤i<Z−c and from the barrel shifter if Z−c≤i<Z. An output block shift_out[i] with i>Z can have any value because its value is ignored in the following blocks. Figure 5b illustrates the operation of the FRCS network with Z=8,P=12 and c=3. The control signal $FRCS_{ctrl}$ is defined as follows:

(2) $$FRCS_{ctrl}\left[i\right]=\left\{\begin{array}{cc} 0 & \;0\leq i<5,\\ 1 & \;5\leq i<8. \end{array}\right.$$

### 3.3. Fine-Coarse Structure for Circular-Shift Network

The fine-coarse structure for circular-shift network is designed to support different block sizes, in which the maximum block size is defined by *P*. Let $Z_{p}$ denotes the GCD between all block sizes, and $Z_{m}=P/Z_{p}$. To support multi circular shifts with low complexity, the proposed low-complexity MSCS network for 5G NR LDPC decoders is decomposed into two parts: the fine part and the coarse part. More precisely, the MSCS network architecture comprises of $Z_{m}$$Z_{p}$-input pre-rotators, plus an additional stage of $Z_{p}$$Z_{m}$-input main rotators. Let *Z* denotes the block size of the circular-shift message and *c* denotes the circular-shift value which is distributed over the range from 0 to Z−1. The fine-coarse structure decomposes the circular shift into a pre-circular shift $c_{p}$ and a main circular shift $c_{m}$. The circular-shift amount *c* can be described by the sum of two terms as $c=c_{p}+c_{m}\times Z_{p}$, where $0\leq c_{p}<Z_{p}$. In the fine-coarse structure, the output data are generated in two steps, which are listed below:

- *Step 1. Pre-rotator*: Each sub-block of $Z_{p}$ input data are processed by a pre-rotator subnetwork to generate the pre-circular shift by performing $c_{p}$ shift operation.
- *Step 2. Main rotator*: The *k*-th ouput datum of each pre-rotator network is distributed into the *k*-th main rotator subnetwork, where $0\leq k<Z_{p}$. The main circular shift is generated by performing $c_{m}$ shift operation on each main rotator subnetwork. To overcome the inside-group rotation at the pre-rotator network, the cross-group movement is performed by the $c_{m}+1$ circular shift at the *k*-th main rotator subnetwork where $k+c_{p}\geq Z_{p}$.

**Figure 6:**
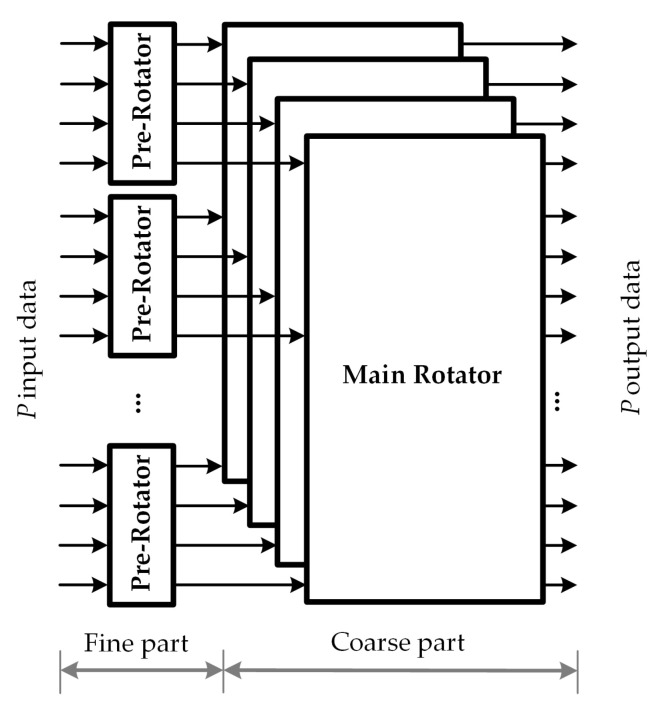
Fine-coarse structure.

As shown in Figure 6, the fine-coarse structure has two components, i.e., the pre-rotator network, and the main rotator network, which corresponds to step 1, and 2 described above. In this structure, the support of multiple sizes is achieved by rearranging the system into smaller subnetwork with fixed block sizes.

### 3.4. Proposed Low-Complexity Multi-Size Circular-Shift Network

**Figure 7:**
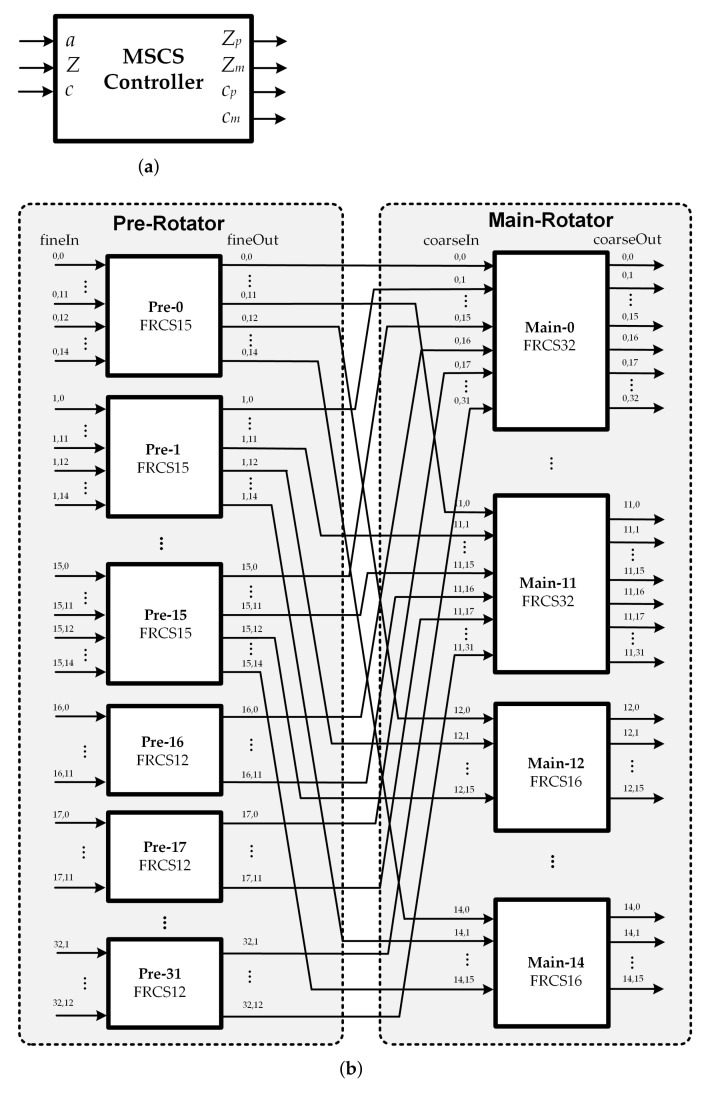
Proposed MSCS network structure for 5G NR LDPC decoders: (**a**) MSCS controller; (**b**) Two stages of the sub-MSCS network.

The principal of our proposed low-complexity MSCN structure for 5G NR LDPC decoders is based on the fine-coarse structure and the forward routing network. The design target of the proposed architecture is focused on multi-size message-passing network with lower complexity. In general, the fine-coarse structure decomposes the system into smaller subnetworks with fixed block sizes. The block size of the subnetworks is determined by the GCD of all supported block sizes. For 5G NR LDPC codes, the block sizes vary over a very large range from 2 to 384. In this case, the MSCN structure would be made of 192 2-input pre-rotators, plus an additional stage of 2 192-input main rotators as the GCD is 2. When the size of the submatrix is 384, this approach reduces the hardware complexity compared with the direct implementation using 384 384-to-1 MUXs. However, it still suffers from significant hardware overhead. Therefore, the same technique is not directly applicable in the case of 5G NR LDPC codes. There is a need to accommodate all 51 block sizes while achieving lower complexity. To overcome the drawback, a novel MSCS network architecture based on fine-coarse structure, in which the subnetworks are reconfigurable, is presented in this section. Figure 7 illustrates the proposed low-complexity MSCS network structure. As shown in Figure 7b, the proposed structure can be constructed from two stages of the sub-MSCS network in which FRCS structure is conducted. First, denote the maximum block sizes of the MSCS network is *P*, the size of the submatrix for the circular shift is *Z*, and the value of the required circular shift is *c*. For this fine-coarse structure, $\left(P_{p},Z_{p},c_{p}\right)$ and $\left(P_{m},Z_{m},c_{m}\right)$ which are the set of maximum block size, the size of the submatrix for the circular shift, and the circular-shift value for the sub-MSCS network in the pre-rotator and main rotator stage, respectively, are further defined.

Based on the characteristics of the modified lifting sizes *Z* table in Figure 4b, the pre rotator block can be constructed from 32 FRCSs, named as Pre[i], in which the first 16 FRCSs (for 0≤i<16) are with input size $Z_{p}=15$ (FRCS15) and the remaining 16 FRCSs for 16≤i<32 are with input size $Z_{p}=12$ (FRCS12). Similarly, the main rotator is decomposed into 15 FRCSs, denoted as Main[j]. The first to the 12th FRCS (FRCS32) (for 0≤j<12) have the same number of $Z_{m}=32$ input blocks while the remaining 3 FRCSs (FRCS16) only comprise of $Z_{m}=16$ input blocks for 12≤j<15. In this structure, all the control signals can be implemented with small area and determined on-the-fly. The control signal for the proposed MSCS network would be calculated from the values of *P*, *c* and *a*, as illustrated in Figure 7b. Please note that the values of *a* in the modified table in Figure 4b are handled instead of the original ones. The MSCS network controller consists of two parts: a circular-shift sizes generator and a circular-shift amount generator. The values of the circular shift sizes for pre rotator and main rotator subnetworks are defined as follows:

(3) $$\begin{array}{c} Z_{p}=a,\\ Z_{m}=\frac{Z}{a}. \end{array}$$

In the fine-coarse structure, the circular-shift amount *c* can be described by the sum of two terms as $c=c_{p}+c_{m}\times Z_{p}$, where $0\leq c_{p}<Z_{p}$ as mentioned in previous section. Therefore, the circular-shift values for the two rotators can be determined as follows:

(4) $$c_{p}\left[i\right]=c\;\left(\mathrm{mod}\;Z_{p}\right),\;0\leq i<32,$$

(5) $$c_{m}\left[j\right]=\left\{\begin{array}{cc} \frac{c}{Z_{p}} & \;\mathrm{if}\;0\leq j<Z_{p}-c_{p},\\ \frac{c}{Z_{p}}+1 & \;\mathrm{if}\;j\geq Z_{p}-c_{p}. \end{array}\right.$$

The MSCS network concurrently routes all messages through the two-stage network. As first, the pre-circular shift with a size of $c_{p}$ is performed on the *Z* messages, and then the main circular shift with a size of $c_{m}$ is finally performed on the result of the pre-circular shift. First, the input data of size *Z* are evenly distributed among the first $Z_{m}$ pre rotators of the fine part. The rest of the pre rotator stage is ignored. For instance, the first pre rotator Pre[0] takes the first $Z_{p}$ signals from the input messages, the second pre rotator Pre[1] selects the next set of $Z_{p}$ signals, and the last set of $Z_{p}$ signals will be fed into the final pre rotator $Pre[Z_{m}-1]$. Each pre rotator block carries out the circular shift of $Z_{p}$ messages by $c_{p}\left[i\right]$. Before the main circular shift is exploited, the proper outputs from the pre rotator stage are assigned to the inputs of each main rotator block. In this stage, the *j*th $\left(0\leq j<Z_{p}\right)$ main rotator block takes all the *j*th output signal of each pre rotator in previous stage. Similarly, each main rotator block performs the circular shift on the selected $Z_{m}$-message with the circular-shift value $c_{m}\left[i\right]$, which is defined in Equation (5). To achieve the final circular shift message, each $Z_{m}$-message from the first to the $Z_{p}$th main rotator block is sequentially routed to the output of the MSCS network.

## 4. Implementation Results and Comparison

Table 1 reports a resource breakdown of the proposed MCSN for 5G NR LDPC decoder. As can be seen from Table 1, the proposed MCSN requires 972,256 adders/subtractors, 483,092 comparators, 732,228 multiplexers and 482,448 logic shifters. The number of adders is about 85% of the number of subtractors. The greater comparator is the majority of the comparators.

To confirm the efficiency of our solution, we have compared the conventional RIP structure [23], the fine-coarse architecture with MUXs network [16], the fine-coarse structure with RIP network [24] with our proposed MCSN network for multi-size message passing network of the 5G LDPC decoder where the full lifting set *Z* is given by {2, 3, 4, 5, 6, 7, 8, 9, 10, 11, 12, 13, 14, 15, 16, 18, 20, 22, 24, 26, 28, 30, 32, 36, 40, 44, 48, 52, 56, 60, 64, 72, 80, 88, 96, 104, 112, 120, 128, 144, 160, 176, 192, 208, 224, 240, 256, 288, 320, 352, 384}. The proposed MCSN network structures are synthesized using the TSMC 65-nm CMOS technology. The ASIC post synthesis implementation results on are reported in Table 2.

As can be seen from Table 2, the proposed MCSN structure occupies an area of 0.301 mm${}^{2}$ and works at a high frequency of 580 MHz. Since the proposed work and other architectures are implemented with different parameters such as maximum shift size *P*, CMOS technology, and the quantization bits, it is necessary to apply performance normalization to 65-nm CMOS technology for a fair comparison. Specifically, the normalization method presented in [25] is used. We also consider the maximum shift size *P* and the quantization bits in the normalization. From the normalized results, it is confirmed that the proposed solution helps reduce the complexity in comparison to the works introduced in [16,23,24]. Specifically, the area reduction from the RIP structure [23], the fine-coarse architecture with MUXs network [16], and the fine-coarse structure with RIP network [24] are 56.75%, 18.87%, and 49.75%, respectively. The proposed MCSN can be well operated at the high frequency of 580 MHz. Therefore, the proposed MCSN structure is a promising solution to reduce the complexity of the QC-LDPC decoders for 5G NR.

## 5. Conclusions

In this paper, a novel reconfigurable message-passing network architecture has been proposed for 5G NR QC-LDPC decoders. The proposed MCSN structure using fine-coarse approach with low complexity is capable of supporting 51 different submatrix sizes of the 5G NR standard and exhibits low complexity. Simulation results prove that the proposed MCSN structure requires small area over its predecessors. Therefore, the proposed design is appropriate for applications with multimode QC-LDPC decoders in 5G standard requirements.

## Figures and Tables

**Table 1:** Resource Breakdown of Multi-Size Circular-Shift Network for 5G NR LDPC Decoders.

Components		Resource Consumption
Adders/Subtractors	Adders	445,970
	Subtractors	526,286
Comparators	Lessequal Comparators	2070
	Greater Comparators	481,022
Multiplexers		732,228
Logic Shifters		482,448

**Table 2:** ASIC Implementation Results of Multi-Size Circular-Shift Network for 5G NR LDPC Decoders.

	Proposed	RIP [23,24]	FC + MUXs [16]	FC + RIP [24]
Maximum size *P*	384	96	96	96
CMOS technology	65-nm	32/28-nm	130-nm	32/28-nm
Message bits	8-bit	8-bit	6-bit	8-bit
Frequency (MHz)	580	637	500	649
Norm. Frequency (MHz)	580	314	1000	320
Area (mm${}^{2}$)	0.301	0.042	0.278	0.036
Norm. Area (mm${}^{2}$)	0.301	0.696	0.371	0.599
